# Smad4-deficient T cells promote colitis-associated colon cancer *via* an IFN-γ-dependent suppression of 15-hydroxyprostaglandin dehydrogenase

**DOI:** 10.3389/fimmu.2022.932412

**Published:** 2022-08-15

**Authors:** Sung Hee Choi, Alex Y. Huang, John J. Letterio, Byung-Gyu Kim

**Affiliations:** ^1^ Case Comprehensive Cancer Center, Case Western Reserve University, Cleveland, OH, United States; ^2^ Department of Pediatrics, Case Western Reserve University School of Medicine, Cleveland, OH, United States; ^3^ The Angie Fowler Adolescent and Young Adult Cancer Institute, University Hospitals (UH) Rainbow Babies and Children’s Hospital, Cleveland, OH, United States

**Keywords:** IFN-gamma, Smad4, CD4 effector T cell, 15-PGDH, colitis-associated colon cancer

## Abstract

Immune cells and the cytokines they produce are important mediators of the transition from colitis to colon cancer, but the mechanisms mediating this disease progression are poorly understood. Interferon gamma (IFN-γ) is known to contribute to the pathogenesis of colitis through immune modulatory mechanisms, and through direct effects on endothelial and epithelial homeostasis. Here we explore whether IFN-γ influences tumor progression by expanding the effector memory T cells (T_EM_) population and restricting the expression of tumor suppressors in a preclinical model of spontaneous colitis-associated colorectal cancer (CAC). We show that IFN-γ expression is significantly increased both in the T cells and the colonic mucosal epithelia of mice with a T cell-restricted deletion of the TGF-β intermediate, SMAD4 (Smad4^TKO^). The increase of IFN-γ expression correlates with the onset of spontaneous CAC in Smad4^TKO^ mice by 6 months of age. This phenotype is greatly ameliorated by the introduction of a germline deletion of IFN-γ in Smad4^TKO^ mice (Smad4^TKO^/IFN-γ^KO^, DKO). DKO mice had a significantly reduced incidence and progression of CAC, and a decrease in the number of mucosal CD4^+^ T_EM_ cells, when compared to those of Smad4^TKO^ mice. Similarly, the colon epithelia of DKO mice exhibited a non-oncogenic signature with a decrease in the expression of iNOS and p-STAT1, and a restoration of the tumor suppressor gene, 15-hydroxyprostaglandin dehydrogenase (15-PGDH). *In vitro*, treatment of human colon cancer cells with IFN-γ decreased the expression of 15-PGDH. Our data suggest that Smad4-deficient T cells promote CAC through mechanisms that include an IFN-γ-dependent suppression of the tumor suppressor 15-PGDH.

## Introduction

Colorectal cancer (CRC) is the third most commonly occurring cancer and is the second most common cause of cancer-related deaths in the world (1, 2). Globally the incidence of CRC is increasing and it is expected to increase to more than 2.2 million new cases and 1.1 million cancer deaths by 2030 (2). Inflammatory bowel disease (IBD), including Crohn’s disease (CD) and ulcerative colitis (UC), is a chronic, progressive inflammatory condition of the gastrointestinal tract (3–5). Patients with IBD have a significantly higher risk for the development of CRC compared with the colitis-free population, and this type of cancer is known as “colitis-associated colorectal cancer (CAC)”.

Although the exact mechanism linking chronic inflammation to carcinogenesis remains unclear, overproduction of inflammatory cytokines and trafficking of effectors leukocytes into the intestinal mucosal microenvironment are thought to be major initiators of the disease process (6). In the azoxymethane/dextran sulphate sodium (AOM/DSS) experimental model of CAC, RAG1-deficient mice that do not have B and T cells did not develop tumors even in the presence of colitis (7). These results indicate that lymphocytes are required to promote tumor growth in the context of colitis. Furthermore, both CD and UC are characterized by an increased mucosal infiltration and activation of CD4^+^ T lymphocytes (8). Expanded and dysregulated lymphocytes are critical in driving gut inflammation, with increased production of inflammatory cytokines. Under healthy conditions, there is a tightly controlled balance between pro-inflammatory mediators (e.g., TNF-α, IL-1β, IL-6, IFN-γ) and anti-inflammatory mediators (e.g., IL-10, TGF-β) (9). In IBD, such balance is lost, and uncontrolled intestinal inflammation is characterized by an overproduction of inflammatory cytokines. These pro-inflammatory cytokines promote epithelial cell proliferation, survival and metastasis through their capacity to activate the transcription factors Stat1, Stat3 and NF-κB (10–12). Their presence has also been associated with changes in intestinal epithelial cell (IEC) homeostasis under certain conditions, but the mechanisms underlying this process are not well characterized.

One of the most highly upregulated cytokines in IBD and mouse models of intestinal inflammation is IFN-γ (13). As a proinflammatory cytokine, IFN-γ is one of the main cytokines identified in the inflamed mucosa of IBD patients, and especially in the patients with CD (14, 15). Robust production of IFN-γ is also seen in the colon of DSS-treated mice (16), and neutralization antibodies against IFN-γ significantly ameliorate chronic intestinal inflammation in the DSS-induced mouse model of colitis (17). Moreover, IFN-γ-deficient mice did not develop DSS-induced colitis (18). Together, these studies suggest that IFN-γ is a key cytokine that is crucially involved in the induction and progression of IBD. Binding IFN-γ to the IFN-γ receptor activates the Janus kinase (JAK)-signaling transducer and activator of transcription (STAT) signaling pathway. Activated JAKs induces STAT dimerization and subsequently expression of different target genes involved in various biological processes (19, 20).

Transforming growth factor-beta (TGF-β) is a pleiotropic cytokine with important functions for the maintenance of immune homeostasis and is one of the key molecules regulating epithelial cell biology and immunity in the gut (21). Inadequate TGF-β signaling in T cells has been implicated in the pathogenesis of IBD and CAC (22, 23). Studies using normal colonic biopsies showed that TGF-β blockade downregulated T cell apoptosis, and induced a significant increase in inflammation (24). Mice deficient for TGF-β1 have a dramatic phenotype, and develop multiple inflammatory changes throughout the body leading to death within 3 weeks of life (25). TGF-β binds to TGF-β receptor 2 (TGFBR2) to activate TGFBR1/2 complex and promotes phosphorylation of Smad2 and Smad3. Phosphorylated Smad2/3 proteins heterodimerize with Smad4 to translocate to the nucleus, where they regulates target genes expressions (26). We showed that mice with a T cell-restricted loss of the *SMAD4* gene (Smad4^TKO^) develop chronic inflammation in the intestinal mucosa, a phenotype associated with spontaneous CAC (27), and the progression to CAC is accelerated by CD4^+^ effector memory T (T_EM_) cells (28). These results confirm that immune cells and the cytokines they produce are important mediators of the transition from colitis to colon cancer, but the mechanisms mediating this disease progression remain to be elucidated.

The tumor suppressor 15-hydroxyprostaglandin dehydrogenase (15-PGDH) is an enzyme responsible for the degradation of PGE2 into an inactive metabolite (29). 15-PGDH is highly expressed in normal colonic mucosa, but 15-PGDH expression is lost in CRC cells (30), an event directly linked to disease progression. Recent reports have explored the efficacy of 15-PGDH as a potential antitumor agent against colon cancer (31) and showed that loss of 15-PGDH has a causal effect in development of colon cancer in the Smad4^TKO^ model (32). The synthetic triterpenoid, CDDO-Me, suppressed colitis-associated colon cancer by inducing 15-PGDH expression (32). While TGF-β induces the expression of 15-PGDH in colon epithelial cells in a Smad3-dependent manner (32, 33), inflammatory cytokines suppresses 15-PGDH expression in IBD (34).

Here we report that in the absence of TGF-β signaling in T cells, the production of proinflammatory cytokines is significantly increased and IFN-γ expression is down-regulated through a Smad4-dependent mechanism. *In vitro* IFN-γ treatment suppresses tumor suppressor 15-PGDH in cultured colon cancer cells. Smad4^TKO^ mice exhibit mucosal epithelial hyperplasia that is accompanied by increased inflammation in colonic mucosa and a significant reduction in the expression of 15-PGDH in colon epithelial cells. Germline deletion of IFN-γ in Smad4^TKO^ mice (Smad4^TKO^/IFN-γ^KO^, DKO) significantly reduces the incidence and progression of CAC *via* a decrease in the population of CD4^+^ T_EM_ cells and a restoration of 15-PGDH expression in colon epithelia, when compared to Smad4^TKO^ mice. These data demonstrate the direct link between alterations of TGF-β signaling in T cells and malignant transformation in epithelial cells in the gastrointestinal tract, through an IFN-γ-dependent alteration in the expression of the tumor suppressor 15-PGDH.

## Methods and materials

### Antibodies

Anti-phospho-Stat1 (Tyr701) (58D6), anti-phospho-Stat3 (Thyr705) (D3A7), anti-phospho-iκB (14D4), anti-iNOS (D6B6S), and anti-PD-L1 (D5V3B) were purchased from Cell Signaling. Anti-CD3, anti-CD28, anti-CD25, anti-CD44, anti-CD62L, anti-IFN-γ, IL-6, and anti-TNF-α were purchased from BD Biosciences. Anti-Foxp3 antibody was purchased from eBioscience.

### Animals

T cell-restricted deletion of the *SMAD4* gene (Smad4^co/co;Lck-cre^, Smad4^TKO^) in mice has been described previously (27, 32). The model characterized by germ line deletion of IFN-γ (IFN-γ^KO^) was purchased from The Jackson Laboratory. To generate mice deficient for both IFN-γ germ line and for Smad4 in the T cell lineage only, IFN-γ^KO^ mice were crossed with Smad4^TKO^ mice. The resulting F1 heterozygotes were then bred to generate all genotypes, including Smad4^TKO^/IFN-γ^KO^ (DKO) mice. Mice were housed in a specific pathogen-free facility. All animal experiments were performed in accordance with institutional guidelines and with approval of the Institutional Animal Care and Use Committee at Case Western Reserve University.

### Assessment of neoplasia and colitis

The colon was excised from the ileocecal junction to the anal verge, flushed with phosphate-buffered saline (Invitrogen) and opened longitudinally. Gross examination was performed to evaluate tumor size and number and to measure colon length and colon weight. The colon length to colon weight ratio was measured to assess thickening of the intestinal mucosa. The incidence (defined as number of mice with tumors/total mice in the group), the mean tumor size ± standard deviation, and the mean number of tumors/mouse ± standard deviation were calculated for each group. Tumor size was determined by image analysis using imaging software (ImageJ). Images were taken with a scale bar and lengths were measured in pixels and correlated to the known distance in scale bars. Colon tumors as well as colonic tissues were processed for histopathological evaluation and further biochemical analyses.

### Nitrite assay

Serum Nitric oxide (NO) levels were measured by photometric analysis by using a nitrite/nitrate assay kit (Cayman Chemical), according to manufacturer’s instructions.

### Quantitative RT-PCR analysis

Colon mucosa was obtained from scrapings of full-length colon and total RNA was isolated using Trizol reagent (Invitrogen). For reverse transcription-PCR (RT-PCR), cDNA was synthesized using a High Capacity cDNA synthesis kit (Applied Biosystems). Quantitative RT-PCR was performed using BioRad CFX96 Real-Time System C1000 Thermal Cycler. The expression of target genes was normalized to expression of housekeeping gene β-actin. The relative gene level was expressed as 2^-ΔΔCt^, in which ΔΔCt equals ΔCt of the experimental sample (Smad4^TKO^, IFN-γ^KO^ or DKO mouse sample) minus ΔCt of the control sample (WT mouse sample).

### Cell cultures, transient transfection, and luciferase assay

The FET human colon carcinoma cells were cultured in MEM (Invitrogen) with 10% fetal bovine serum (Germini) and glutamine (2 mg/ml) at 37 °C in 5% CO_2_. FET cells were seeded in 12-well plates at 1 x 10^5^ per well as triplicates and transiently transfected with 0.2 mg of SBE promoter vector or 2.5 kb 15-PGDH –PGL promoter vector and 20 ng of CMV-renilla using LipofectAMINE Plus as transfection agent according to the manufacturer’s instructions (Invitrogen). Approximately 24 hrs past transfection, cells were treated with IFN-γ (20 ng/ml) or IL-6 (20 ng/ml) for 24 hrs in medium. Luciferase activity was measured using Promega Dual Luciferase Assay Kit (Madison, WI) and a ML3000 Microtiter Plate Luminometer. Data shown represent the mean of three independent experiments. For IFN-γ promoter luciferase activity, pCMV5 plasmid or pCMV5-Smad4 plasmid were transfected one day before transfection of 3.6 Kb IFN-γ promoter in 293T, Jurkat or primary murine T cells and 24 hrs after IFN-γ-promoter transfection, luciferase activity was measured. For T cell activation, T cells were plated on the plate coated with anti-CD3/CD28.

### Western blotting

For Western blot, colon mucosa was obtained from scrapings of full-length colon and lysed by incubation in lysis buffer (150 mM NaCl, 20 mM Tris-Cl, pH 7.5, 1 mM PMSF, 1 mM Na3VO4, 25 mM NaF, 1% aprotinin, 10 μg/ml leupeptin) on ice for 30 min. 20 μg aliquots of proteins were separated by electrophoresis in 10% SDS/PAGE minigels and transferred to nitrocellulose membrane (Invitrogen). Following blocking, membranes were incubated in buffer containing the primary antibody, followed by washing and incubation for 1hr at room temperature with horseradish peroxidase-conjugated secondary antibodies. Immunostaining was visualized by ECL.

### Histology

For hematoxylin and eosin staining (H&E), excised colons were washed with PBS and fixed in 10% formalin. Samples were embedded in paraffin wax, sectioned, stained with H&E, and examined by light microscopy. For immunohistochemistry (IHC), slides were deparaffinized and rehydrated and heat-induced epitope retrieval was performed prior to blocking with Peroxidazed 1 (BioCare, PX968) and Rodent Block M (BioCare, RBM961). Slides were incubated with primary antibodies, CD3 and PD-L1 for one hour at room temperature. Antibodies were detected using Rabbit-on-Rodent HRP polymer (BioCare, RMR622) and visualized using Betazoid DAB chromogen kit (BioCare, BDB2004). The percentage of CD3 positive cells were assessed on six randomly selected field using digital eyepiece.

### Colon lamina propria cells

LP immune cells were isolated using Mouse Lamina Propria Dissociation Kit (Miltenyi Biotec). Colon sections were washed clean, cut into small pieces, and incubated with 5 mM EDTA, 1 mM DTT (MilliporeSigma), and 5% FBS in HBSS buffer (Thermo Fisher Scientific) for 20 minutes. Then tissue was collected into gentle MACS C tube (Miltenyi Biotec) and dissociated to single cells using gentleMACS Dissociator (Miltenyi Biotec).

### FACS analysis

Cell suspensions were prepared from spleens or colon lamina propria by filtering through nylon mesh (40-µm diameter). Erythrocytes were lysed using ACK lysis buffer (BioWhittaker) and cells were washed twice in RPMI 1640 supplemented with 10% heat-inactivated FBS, 50 µM 2-ME, penicillin, and streptomycin (Invitrogen). Viable cells were counted using trypan blue exclusion on a hemocytometer. All Antibodies used in FACS analyses were purchased from BD Pharmingen. Pan T cells were purified from spleen and lymph node using a Pan T Cell Isolation Kit (Miltenyi Biotec) according to the manufacturer’s instructions (purity greater than 95%).

### Intracellular cytokine staining and regulatory T cell induction

For intracellular staining for cytokines, lymphocytes from spleen and colon lamina propria were activated with plate-bound anti-CD3 and anti-CD28 antibodies for 48 hrs and re-stimulated with PMA and ionomycin for the last 5 hrs in the presence of Golgi stop solution prior to washing and staining with antibodies for CD4 and CD8. Intracellular staining for IFN-γ, IL-6 and TNF-α was performed using an intracellular staining kit (BD Biosciences) according to the manufacturer’s instructions. For inducible regulatory T cell assay, splenocytes from each genotype were activated *in vitro* by plate-bound anti-CD3 and anti-CD28 antibodies in 24-well plates in the absence or presence of TGF-β for 72 hrs. The cells were harvested, washed with PBS, stained with anti-CD4 and CD8 antibodies, and stained for intracellular Foxp3 using a Foxp3 staining Kit (eBioscience) according to the manufacturer’s instructions.

### Statistical evaluation

Data are expressed as means ± SE. Statistical significance was determined by 1-way ANOVA with Tukey–Kramer Multiple Comparisons Test. The Fisher’s Exact Probability test was used for comparison of the incidence of lesions between the two groups. Statistical significance was accepted to be a *p*-value less than or equal to 0.05, with *p<0.05, **p<0.01, ***p<0.001.

## Results

### Deficiency of Smad4 in T cells drives a colitis-associated cancer phenotype with an increase in effector memory T cells and a decrease in regulatory T cells

**Figure 1:**
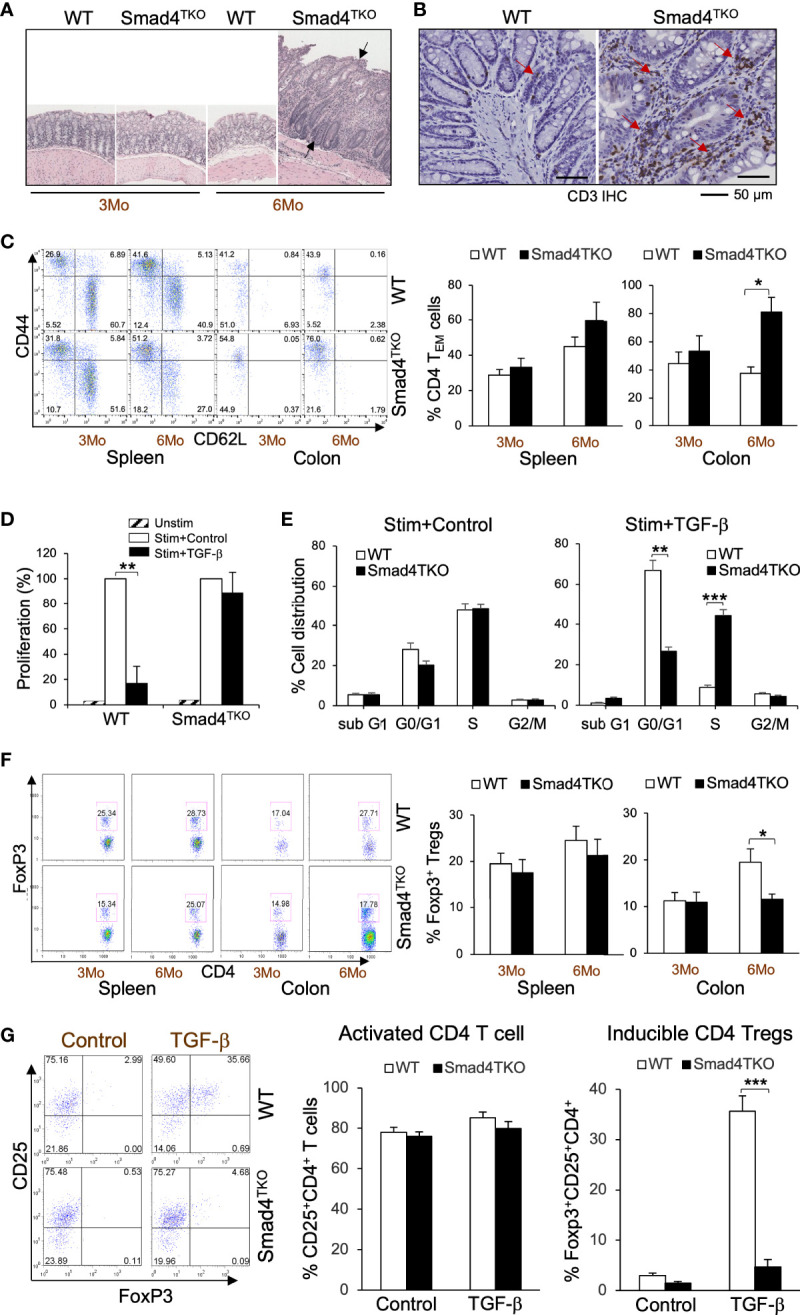
Smad4 deletion in T cell leads inflammation-associated colon cancer with an increase in effector memory T cell population and a decrease in natural regulatory T cell (nTreg) population. **(A)** Hematoxylin and eosin (H&E) staining of the colon of wild type and Smad4^TKO^ mice at 3 and 6 months of age. **(B)** Immunohistochemistry (IHC) staining for CD3 in colons of each genotype at 6 months of age. Scale = 50μm. **(C)** Analysis of effector memory markers (CD44^High^CD62L^Low^) in CD4^+^ T cell compartment of spleen and colon lamina propria of WT and Smad4^TKO^ at 3 and 6 months of age (n=3-5). **(D)** Analysis of CD4 T cell proliferation suppressed by TGF-β. Cells from Spleen per genotype at 2 months of age (n=3) were collected and incubated with anti-CD3/CD28 for 72 hours with or without TGF-β (2 ng/ml) and the proliferation of CD4 T cells was measured by H^3^ thymidine incorporation. **(E)** Cell cycle analysis of CD4 T cells per genotype (n=3). CD4 T cells from panel D were incorporated with BrdU for the last 16 hrs incubation, harvested, intracellularly stained with anti-BrdU and 7AAD. **(F)** Analysis of natural regulatory T (nTreg) cell population in CD4^+^ T cell compartment from spleen and colon lamina propria of WT and Smad4^TKO^ at 3 and 6 months of age (n=3-5). Splenocytes and colon lamina propria cells per genotype at 3 and 6 months of age were collected, stained on cell surface with antibody for CD4 and intracellularly with Foxp3 antibody, and analyzed on CD4^+^ T cells by FACS. **(G)** Analysis of TGF-β-induced Treg (iTgreg) population in CD4^+^ T cell compartment of each genotype. Cells from Spleen per genotype at 2 months of age (n=4) were collected and incubated with anti-CD3/CD28 for 72 hours with or without TGF-β (2 ng/ml) and intracellularly stained with Foxp3 antibody. Error bars indicate S.E.; *p < 0.05, **p < 0.01, ***p < 0.001.

We previously established a novel murine model of colitis-associated colorectal cancer (CAC) through a T cell lineage-restricted deletion of the Smad4 gene in mice (27). In this model, selective loss of Smad4-dependent signaling in T cells (Smad4^co/co;Lck-cre^, Smad4^TKO^) leads to spontaneous mucosal inflammation, and epithelial cancers throughout the gastrointestinal tract. Smad4^TKO^ mice develop CAC, with the majority of tumors arising after 6 months of age (**Figure 1A**, black arrows), and with an increase in mucosal T lymphocytes as enumerated by CD3 immunoreactivity (**Figure 1B**, red arrows). In the mucosa of Smad4^TKO^ at 6 months of age, the population of CD4^+^ effector memory T (T_EM_) cells (CD44^High^CD62L^Low^) was significantly increased (78.74%) compared to wild type mice (35.29%) (**Figure 1C**), whereas no difference was detected in the spleen. TGF-β is a known inhibitor of the proliferation of T cells (22, 35, 36), with TGF-β treatment of T cells leading to cell cycle arrest, typically in the G1 phase (37). In a T cell proliferation assay, TGF-β significantly inhibited proliferation of CD4 T cells from the spleen of WT but failed to inhibit proliferation in the Smad4^TKO^ (**Figure 1D**). These results were supported by cell cycle analysis using BrdU incorporation. As expected, stimulation of T cells increases the percentage in S phase of cell cycle, with an associated decrease in the percentage in G1 phase, an effect that is blocked by TGF-β treatment in WT CD4 T cells, but not in Smad4^TKO^ CD4 T cells (**Figure 1E**, **Supplementary Figure 1A**). In addition to observed increases in T_EM_, the population of Foxp3^+^CD4^+^ regulatory T (Treg) cells in the colon of Smad4^TKO^ was decreased (17.74%) compared with that of WT (27.71%) while there were no significant changes in the splenic Treg population (**Figure 1F**). In an inducible Treg (iTreg) assay, exogenous recombinant TGF-β induces the expression of Foxp3 in naïve CD4^+^ T cells of spleen from WT mice but fails to induce Foxp3 expression in naïve CD4^+^ T cells isolated from Smad4^TKO^ mice (**Figure 1G**), indicating there exist both a decrease in the total Treg population and a deficiency of iTreg induction in the Smad4^TKO^ mice. These results indicate that the CD4 T_EM_ cells are associated with the pathogenesis of CAC in Smad4^TKO^ mice.

### IFN-γ directly inhibits the expression of 15-PGDH in colon carcinoma cells and is more highly expressed in the absence of Smad4 in T cells

**Figure 2:**
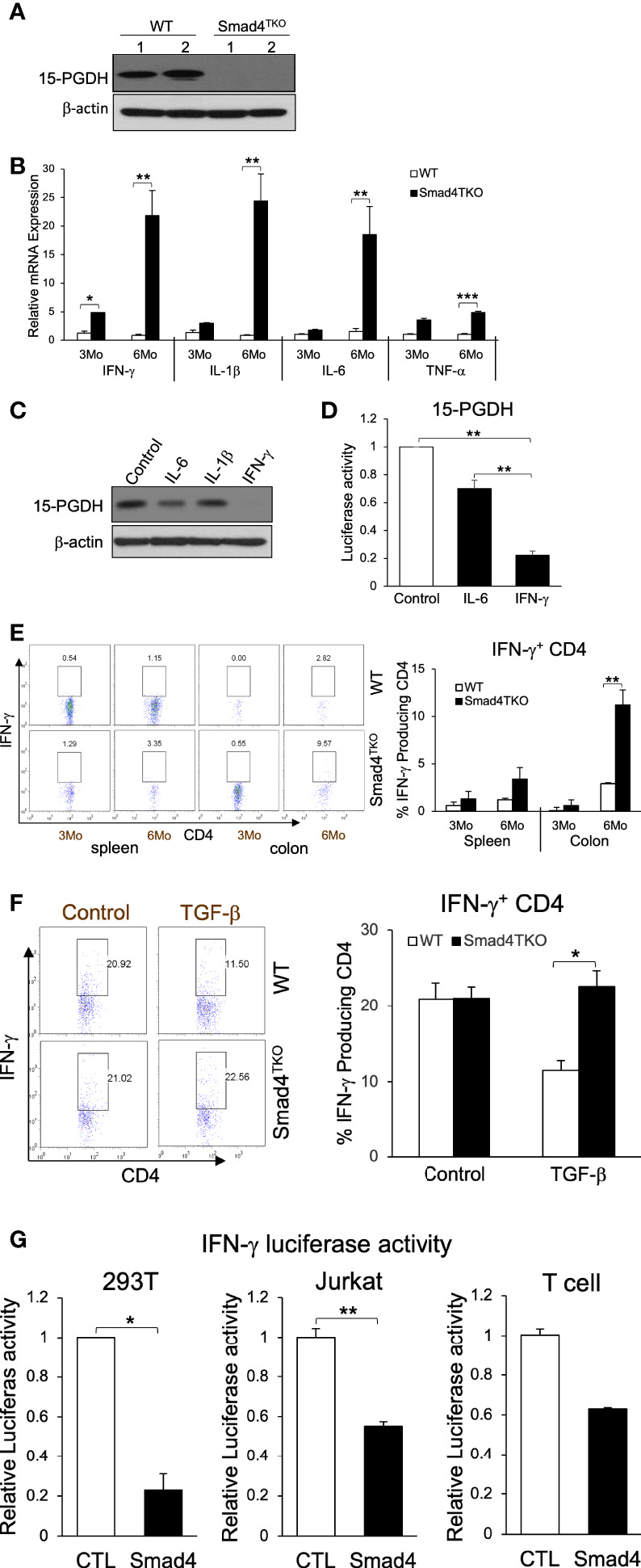
IFN-γ down-regulates 15-PGDH expression in colon carcinoma cells and is inhibited by Smad4-dependent TGF-β signaling in T cells. **(A)** Protein expression of 15-PGDH was examined in the colon of WT and Smad4^TKO^ mice at 6 months of age by Western blot analysis. **(B)** Relative mRNA expression of pro-inflammatory cytokines (IFN-γ, IL-1β, IL-6 and TNF-α) were measured by real time PCR in colon mucosa of WT and Smad4^TKO^ at 3 months and 6 months of age (n=4-6). **(C)** The effects of pro-inflammatory cytokines on 15-PGDH protein expressions in FET colon carcinoma cells. FET cells were treated with pro-inflammatory cytokines including IL-6 (20ng/ml), IL-1β (5ng/ml) and IFN-γ (20ng/ml) for 24 hrs and harvested for Western blot analysis to determine effects of these inflammatory cytokines on 15-PGDH protein expression.**(D)** 15-PGDH promoter activity in FET cells stimulated with IFN-γ (20ng/ml) or IL-6 (20ng/ml) for 24 hrs was measured by using a dual luciferase assay. IFN-γ suppressed 15-PGDH promoter luciferase activity. Results are representative of three different experiments. Bars, S.E. n=4, **p < 0.01 compared with vehicle control group. **(E)** Analysis of effector CD4^+^ T cells expressing IFN-γ of each genotype. Splenocytes and cells from colon lamina propria each genotype at 3 months and 6 months of age (n=5) were collected, stained on cell surface with antibody for CD4 and intracellularly with IFN-γ antibody, and analyzed on CD4^+^ T cells by flow cytometry. **(F)** TGF-β suppression of IFN-γ in WT CD4 T cells, not in Smad4^TKO^ CD4 T cells. CD4 T cells from Spleen each genotype at 2 months of age (n=3) were collected and incubated with anti-CD3/CD28 for 72 hours with or without TGF-β (2 ng/ml), intracellular stained with IFN-γ and analyzed on CD4 T cells by flow cytometry. **(G)** Overexpression of Smad4 suppressed IFN-γ promoter activity. Human IFN-γ promoter was transfected in 293T, Jurkat and T cells and measured luciferase assay by dual luciferase assay. CD3 and CD28 were coated in the plate before plating T cells (n=3). Error bars indicate S.E.; *p < 0.05, **p < 0.01 compared with control plasmid transfection.

To investigate the mechanism underlying the link between the increased pathogenic T_EM_ cells and the malignant transformation of colonic epithelium in Smad4^TKO^ mice, we examined the expression levels of tumor suppressors in the colon of Smad4^TKO^ mice. The expression level of the tumor suppressor 15-PGDH was significantly reduced in the colons of Smad4^TKO^ mice, implying that soluble factors from the pathogenic T_EM_ may be responsible (**Figure 2A**). Cytokines including IFN-γ, IL-1β, IL-6 and TNF-α and many chemokines are known to promote inflammation and CAC development through mechanisms that include deregulation of mucosal immune homeostasis and increased epithelial proliferation as well as through the induction of mutations in oncogenes and tumor suppressor genes (10–12, 38). In order to elucidate the mechanisms underlying the decrease in 15-PGDH expression in the colon of Smad4^TKO^ mice, we examined expression levels of inflammatory mediators in the colon of WT and Smad4^TKO^ mice. The mRNA levels of pro-inflammatory cytokines such as IFN-γ, IL-1β, IL-6 and TNF-α were significantly elevated in the colons of Smad4^TKO^ mice at 6 months of age as well as 8 months of age, compared with those of WT mice (**Figure 2B**, **Supplementary Figure 2A**). Notably, among the several proinflammatory cytokines that are greatly increased in the colon of Smad4^TKO^ mice, only IFN-γ completely suppressed the expression of 15-PGDH in FET colon carcinoma cells *in vitro* (**Figure 2C**) (32). The results were confirmed by 15-PGDH luciferase assay (**Figure 2D**). In addition to the increased transcript level of IFN-γ, the population of IFN-γ producing effector T cells in the colons of Smad4^TKO^ mice was significantly increased as compared to that of WT mice (**Figure 2E**, **Supplementary Figure 2B**). We also found that TGF-β decreases the expression of IFN-γ in WT CD4 T cells, but not in Smad4-deficient CD4 T cells (**Figure 2F**). Moreover, we observed that IFN-γ luciferase activity in the 293T and Jurkat cell lines, as well as primary T cells transfected with a vector expressing Smad4 is significantly decreased when compared with those cells transfected with a control vector (**Figure 2G**). These data suggest that Smad4 can directly interfere with the expression of IFN-γ from activated effector T cells, showing the potential mechanisms underlying the ectopic expression of IFN-γ in CD4 T cells from the colons of Smad4^TKO^ mice. Taken together, these data imply that IFN-γ ectopically expressed from Smad4-deficient effector CD4 T cells can suppress 15-PGDH expression, ultimately contributing to acceleration of the development and growth of CAC.

### Germ line deficiency of IFN-γ ameliorates colitis-associated colorectal tumorigenesis in Smad4^TKO^ mice

**Figure 3:**
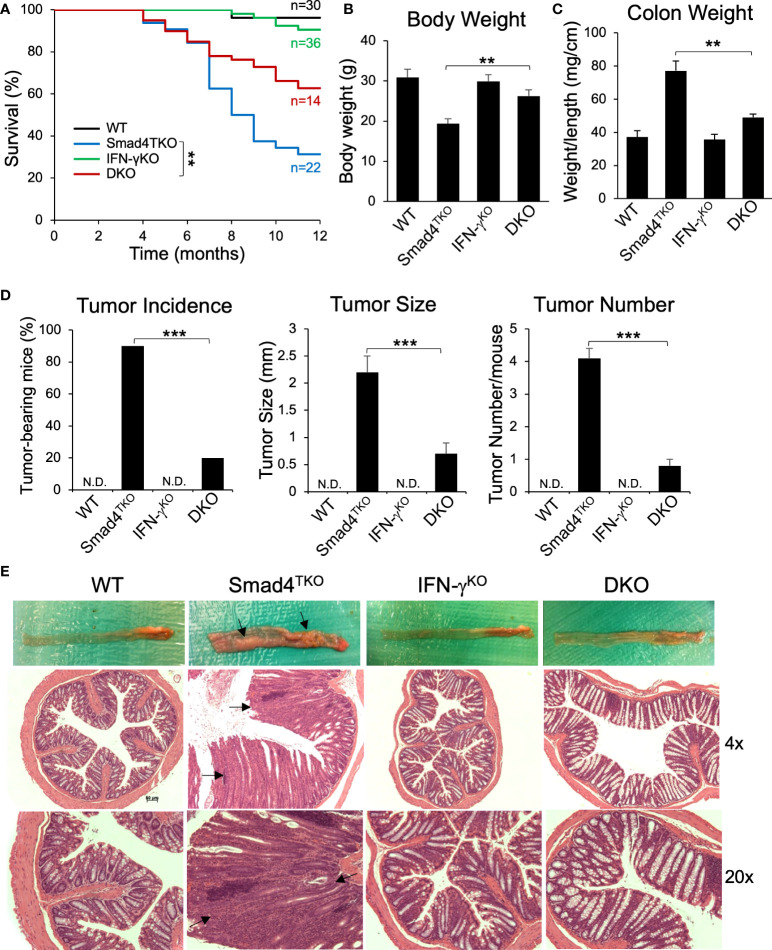
Deletion of IFN-γ in Smad4^TKO^ mice ameliorates colitis-associated colon cancer. **(A)** Survival curves of WT, Smad4^TKO^, IFN-γ^KO^ and Smad4^TKO^/IFN-γ^KO^ (DKO) mice. **(B)** Body weight of each genotype at 8 months of age (n=11). **(C)** Colon weight per length (mg/cm) (n=9). **(D)** Percentage of tumor-bearing mice (n=10), tumor size (n=7) and tumor numbers per mouse at 8 months of age were determined using a digital eyepiece and an imageJ (n=7). **(E)** Gross necropsy and hematoxylin and eosin (H&E) staining of the colon of each genotype at 8 months of age. Paraffin-embedded sections were stained with H&E. Error bars indicate S.E.; **p < 0.01, ***p < 0.001 compared with Smad4^TKO^.

Based on the data described above, we hypothesized that the gain of T cell expression of IFN-γ in Smad4^TKO^ mice undermines the maintenance of both intestinal epithelial and mucosal immune homeostasis. Therefore, to directly demonstrate the contribution of IFN-γ in the pathogenesis of inflammation-driven colon cancer in Smad4^TKO^ mice, we introduced the lineage-restricted *SMAD4* deletion (Smad4^TKO^) onto a genetic background with a germ line *IFN-γ* deletion (IFN-γ^KO^) to generate a ‘double knockout’ (Smad4^TKO^/IFN-γ^KO^, DKO) model. DKO mice harboring the T cell-restricted deletion of the tumor suppressor *SMAD4* and a germ line deletion of proinflammatory cytokine *IFN-γ* ameliorated CAC and inflammatory infiltration of the mucosa as early as 8 months of age, at which point the mortality rate of DKO mice began to decrease compared with the Smad4^TKO^ mice. The survival rate of DKO was 75% at the age of 10 months, compared to 38% for Smad4^TKO^ mice (**Figure 3A**). Clinical features of systemic illness (lethargy, hunched posture, disheveled fur) became evident by 8 months of age in the Smad4^TKO^ mice but not in the DKO mice, and necropsy studies at this age revealed significant gastrointestinal pathology in Smad4^TKO^ mice as a cause of clinical symptoms. The body weight of Smad4^TKO^ mice was decreased, compared with those of WT, IFN-γ^KO^ and DKO mice at 8 months of age (**Figure 3B**). The colons of DKO mice were thinner than that of the Smad4^TKO^ mice (**Figure 3E**) and the significant decrease in colon thickness, as measured by colon weight-to-length ratio, was evident in DKO mice at 8 months of age (**Figure 3C**). Necropsy and mucosal histology showed that deletion of IFN-γ in Smad4^TKO^ mice significantly ameliorates tumorigenesis in the colon at 8 months of age (**Figure 3D**). At this age, tumor incidence (20%) in DKO mice was significantly decreased, when compared with that (90%) in Smad4^TKO^ mice. Average tumor size (0.7 mm) in DKO was significantly smaller than that (2.3 mm) in Smad4^TKO^ mice. Furthermore, tumor multiplicity was less than 1 tumor/mouse in DKO mice, whereas more than 4 tumor/mouse were found in Smad4^TKO^ mice. DKO mice showed a similar phenotype as WT or IFN-γ^KO^, with a delayed disease presentation (12 months) as compared with the Smad4^TKO^ (8 months). These data clearly demonstrate that germ line IFN-γ deletion decelerates CAC development and tumorigenesis in the Smad4^TKO^ mice. Histological analysis of intestinal sections from Smad4^TKO^ mice revealed disrupted villus architecture with regions of epithelial atypia, as well as adenomas and invasive carcinomas as compared to the colon histology of the WT, IFN-γ^KO^ and DKO mice (**Figure 3E**, arrows).

### The CD4^+^ T_EM_ cell population is decreased by IFN-γ deficiency in Smad4^TKO^ mice

**Figure 4:**
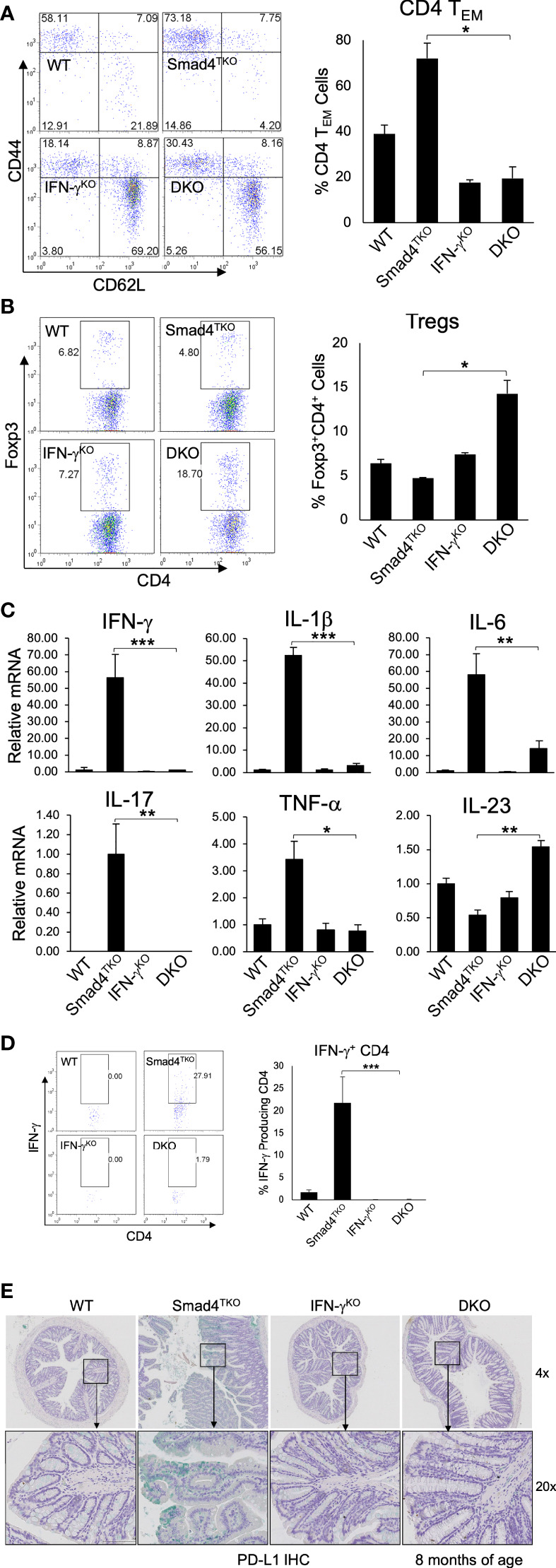
Deletion of IFN-γ in Smad4^TKO^ mice decreases the population of effector memory CD4^+^ cells. **(A)** Analysis of effector memory markers in CD4^+^ T cell compartment in the spleen of each genotype. Spleen cells per each genotype at 8 months of age were collected, stained with antibodies for CD4 and CD8, and analyzed for effector memory markers (CD44^High^ and CD62L^Low^) in CD4^+^ T cell compartment. The bar graphs show the proportion of CD4 T_EM_ cells from the flow cytometry analysis in the left (n=5). **(B)** Natural regulatory T cells in each genotype (n=3-5). **(C)** Relative mRNA expressions of pro-inflammatory cytokines including IFN-γ IL-1β, IL-6, IL-17, TNF-α and IL-23 in colon of WT, Smad4^TKO^, IFN-γ^KO^ and DKO at 6 months (n=5-7). **(D)** Analysis of effector CD4^+^ T cells expressing IFN-γ in the colon of each genotype. Colon lamina propria per genotype were collected, stained on cell surface with antibody for CD4 and intracellularly with IFN-γ antibody, and analyzed on CD4^+^ T cells by FACS. **(E)** Immunohistochemistry (IHC) staining for PD-L1 in colons of each genotype. Error bars indicate S.E.; *p < 0.05, **p < 0.01, ***p < 0.001.

While the data above confirm a role for IFN-γ regulating development of mucosal epithelial malignancy, the decelerated tumorigenesis in the DKO model may also be linked to a more slow expansion of tissue-resident memory T cells, which are known to require TGF-β for their differentiation and accumulate in the mucosa of IBD patients (39, 40). The function of CD4^+^ effector memory T (T_EM_) cells in cancer is complex, and is often dysregulated within the TME (41–43). Abnormal CD4^+^ T_EM_ cell activation and expansion is associated with the development of colitis, which ultimately contributes to the pathogenesis of CAC (28). Although IFN-γ is markedly abundant in memory CD4^+^ T cells, the relevance of IFN-γ activity in T cells and the progression of CAC has not been explored. Thus, we examined the role of IFN-γ in T cell differentiation and function in the Smad4^TKO^ mouse model of spontaneous CAC. We examined the populations of CD4^+^ T_EM_ cells expressing CD44^High^ and CD62L^Low^ in spleen from each genotype at 8 months of age by FACS analysis (**Figure 4A**). The proportion of CD4^+^ T_EM_ cells in DKO mice was diminished when compared with that of Smad4^TKO^ mice, while the proportion of Foxp3^+^ regulatory CD4^+^ T cells was greater (**Figure 4B**). In addition, Smad4 is essential for the induction of iTregs by TGF-β (**Supplementary Figure 3**).

Cytokines including IFN-γ, TNF-α, IL-6 and IL-1β and many chemokines are known to promote inflammation and CAC development through the mechanisms that include deregulation of mucosal immune homeostasis and increased epithelial proliferation as well as through the induction of mutations in oncogenes and tumor suppressor genes (10–12, 38). In order to elucidate the mechanism underlying the pathogenesis of CAC promoted by IFN-γ in Smad4^TKO^ mice, we examined expression levels of inflammatory mediators in the colon of WT, Smad4^TKO^, IFN-γ^KO^ and DKO mice. The mRNA levels of pro-inflammatory cytokines, such as IFN-γ, IL-1β, IL-6, IL-17, and TNF-α were significantly reduced in the colons of DKO mice at 8 months of age, compared with those found in Smad4^TKO^ mice (**Figure 4C**).

We next examined IFN-γ^+^ effector CD4^+^ T cells, which are known to promote genomic instability *via* various mechanisms in the target organ. As expected, the population of effector T cells producing IFN-γ was decreased in DKO mice at 8 months of age, compared with those of Smad4^TKO^ mice (**Figure 4D**). Considering the highly expressed IFN-γ in Smad4^TKO^ colon mucosa as well as the increased IFN-γ secretion observed in CD4^+^ Smad4^TKO^ effector T cells (**Figure 4**), we next investigated immune checkpoint molecules in the colons of each phenotype. IFN-γ is known to induce programed death ligand-1 (PD-L1) expression on tumor cells and immune cells that are also abundant in Smad4^TKO^ colon. We found that IFN-γ deletion in Smad4^TKO^ mice significantly decreases PD-L1 expression in colon mucosa of DKO mice, compared with those of Smad4^TKO^ group (**Figure 4E**, **Supplementary Figure 4A**). Thus, these data suggest that Smad4 deficiency in T cells is associated with the induction of an inflammatory disease state that may confer protection of transformed or malignant epithelial cells from host anti-tumor immune response through mechanisms including the induction of tumor PD-L1 expression. The significance of this observation is supported by data demonstrating elevated mucosal epithelial expression of PD-L1 in IBD patients, and by the observation that pre-existing IBD predicts a risk for severe adverse events in cancer patients treated with immune checkpoint inhibitors (44–46).

### Mucosal inflammation and malignancy in Smad4^TKO^ mice are attenuated by disruption of the IFN-γ – 15-PGDH axis

**Figure 5:**
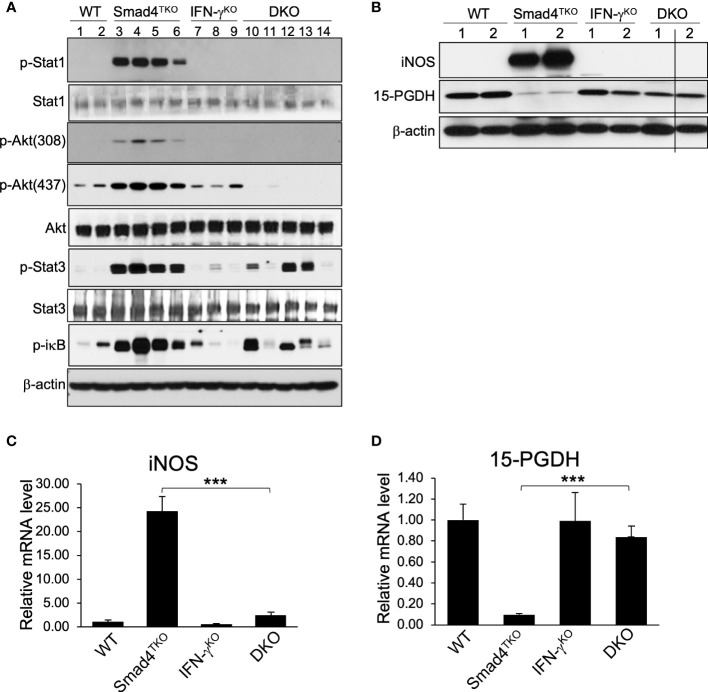
Deletion of IFN-γ in Smad4^TKO^ mice inhibits inflammatory mediators and restores tumor suppressors in colon. **(A)** Protein expression of p-Stat1, Stat1, p-Akt(308), p-Akt(437), Akt p-Stat3, Stat3, p-ikb, and β-actin was measured by Western blot analysis of colon epithelial cell scrapings of each genotype. **(B)** Protein levels of iNOS, 15-PGDH and β-actin in colon of WT, Smad4^TKO^, IFN-γ^KO^ and DKO mice. **(C)** Relative mRNA levels of iNOS and **(D)**15-PGDH were measured by real time PCR in colon mucosa of WT, Smad4^TKO^, IFN-γ^KO^ and DKO mice at 8 months of age (n=4-6). Error bars indicate S.E.; ***p < 0.001.

In order to elucidate the mechanism underlying the pathogenesis of CAC ameliorated by IFN-γ deficiency in Smad4^TKO^ mice, we examined expression levels of inflammatory mediators in the colon of WT, Smad4^TKO^, IFN-γ^KO^ and DKO mice. The mRNA levels of pro-inflammatory cytokines, such as IFN-γ, IL-6, IL-17, TNF-α, and IL-1β were significantly elevated in the colons of Smad4^TKO^ mice at 8 months of age, compared with those of either WT, IFN-γ^KO^ or DKO mice (**Figure 4C**). We observed a correlation between the expression of these inflammatory cytokines and the activation of the intracellular mediators of their response, including NF-κB, Stat1, Stat3 and Akt in the intestinal mucosa in Smad4^TKO^ mice, as assessed by Western blot analysis (**Figure 5A**). The phosphorylation of Stat1 is barely detectable in the colons of DKO mice at 8 months of age, whereas it is significantly increased in Smad4^TKO^ mice. The phosphorylation of Stat3, Akt and iκB was also greatly decreased in colonic mucosal scrapings of DKO mice, compared with those of the Smad4^TKO^ group (**Figure 5A**). Highly increased expression of inducible nitric oxide synthase (iNOS) is a common phenomenon during chronic inflammation. Expression of iNOS protein and mRNA in colonic mucosa was decreased in DKO mice, compared to Smad4^TKO^ mice at this age (**Figures 5B, C**). While iNOS induction is not detected in the colon of WT, IFN-γ^KO^ or DKO mice, it is produced at a great amount in Smad4^TKO^ mice. Moreover, while the tumor suppressor 15-PGDH is significantly suppressed in the colonic epithelia of Smad4^TKO^ mice, the deletion of IFN-γ in Smad4^TKO^ mice completely restored its expression at both the level of mRNA and protein (**Figures 5B, D**). Altogether, these results suggest that IFN-γ ectopically produced from the pathogenic Smad4^TKO^ T_EM_ population induces an inflammatory condition that ultimately leads to the spontaneous initiation and progression of colon epithelial tumorigenesis through an IFN-γ-dependent suppression of the 15-PGDH tumor suppressor.

## Discussion

In this study we have demonstrated a role for IFN-γ production by Smad4-deficient pathogenic T cells in the pathogenesis of inflammation-related CRC in mice. Our data support the finding that IFN-γ abundance accelerates gastrointestinal epithelial malignancy by promoting epithelial cell transformation through suppressed production of the tumor suppressor 15-PGDH in the colonic epithelial cells as well as enhanced production of pro-inflammatory mediators by tissue resident CD4^+^ T_EM_ cells. Our observations in the CAC mouse model resulting from T cell-restricted loss of TGF-β-dependent Smad4 (Smad4^TKO^) were validated by introducing the germ line deletion of the *IFN-γ* gene into Smad4^TKO^ mice (Smad4^TKO^/IFN-γ^KO^, DKO). Utilizing the DKO mouse model, we discovered that the CAC phenotype in Smad4^TKO^ mice is linked to gain of IFN-γ expression in lymphocytes, with significant skewing of the mucosal CD4^+^ T cell repertoire toward an activated, effector memory phenotype. However, other types of cells, such as CD8 T cells, NK cells, and NKT cells cannot be ruled out as a potential important source of IFN-γ expression in this model system. Invariably, the colonic epithelium of Smad4^TKO^ mice exhibited an inflammation-driven oncogenic signature that includes a significant suppression in the expression of 15-PGDH and an elevation in the expression of iNOS, p-Stat1 and p-Stat3.

While the inflammatory function of IFN-γ is well recognized, the current study sheds new light on a unique mechanism through which an abundance in immune cell expression of IFN-γ promotes epithelial carcinogenesis. We discovered that, through direct effects on epithelial cell expression of the tumor suppressor 15-PGDH, IFN-γ directly enhances the initiation and progression of spontaneous epithelial carcinogenesis induced by inflammatory mediators, including iNOS, p-Stat1, p-Stat3 and p-iκB (**Figure 5**). The epithelial-intrinsic mechanisms underlying IFN-γ initiation and progression of CAC include the suppression of the mucosal expression of 15-PGDH. *In vitro* IFN-γ treatment of cultured colon carcinoma cells suppressed 15-PGDH expression (**Figure 2C**). Deletion of IFN-γ gene expression in Smad4^TKO^ mice fully restored the expression of 15-PGDH in the colon which is inversely correlated with colon epithelial carcinogenesis.

15-PGDH expression may be lost in colon epithelial cancers through likely multiple mechanisms, including active suppression of *15-PGDH* gene transcription by proinflammatory cytokines. The loss of 15-PGDH expression may also result from impaired TGF-β signaling (32), a common event in colon and gastric cancers (47). In Smad4^TKO^ mice, expression of 15-PGDH was most likely suppressed in colon epithelial cells as a consequence of the significant increase of IFN-γ secreted during progressive colon inflammation. In mice with DSS-induced colitis, 15-PGDH was completely suppressed in the colonic mucosa (32). This observed reduction of 15-PGDH in Smad4^TKO^ mice was reversible by deletion of IFN-γ, and restoration of 15-PGDH was also accompanied by a reduction in the mucosal inflammatory process. These results also provide evidence of the biological significance of 15-PGDH in colitis and CAC and point to a role for 15-PGDH as a key regulator of epithelial homeostasis during the mucosal inflammatory process.

TGF-β signaling also plays a prominent role in the maintenance of mucosal epithelial and immune homeostasis. Our data suggest that the expansion of a pathogenic effector CD4^+^ T cell population is a direct consequence of a Smad4 deficiency, pointing towards an important mechanism through which Smad4 influences the progression of CAC. We have recently demonstrated a decrease in p27^Kip1^ expression in Smad4 deficient T cells and that p27^Kip1^ deficiency increased the proportion and number of CD4^+^ T_EM_ cells and effector CD4^+^ T cells producing IFN-g in Smad4^TKO^ mice (28). These results are consistent with a previous report that p27^Kip1^ negatively regulates the magnitude and persistence of CD4^+^ T cell memory by promoting apoptosis and contraction of effector CD4^+^ T cells (48). In addition, the possibility that the expansion of effector CD4 T cells could be an indirect consequence of decrement of Tregs cannot be ruled out, as shown in **Figures 1F, G**, especially in an *in vivo* setting. Specifically, this study demonstrates that Smad4 indirectly inhibits the initiation and progression of CAC through suppression of CD4^+^ T cell-mediated mucosal inflammation. Aberrant or dysregulated CD4^+^ T cell memory populations is suspected to contribute to multiple chronic or recurring inflammatory and immune-mediated disorders and to the progression of neoplastic disease (49, 50). Here we show that Smad4 directly decreases the expression of IFN-γ in T cells and that Smad4 deficiency increases the population of CD4^+^ T_EM_ cells and effector CD4^+^ T cells producing IFN-γ in Smad4^TKO^ mice. Importantly, IFN-γ deficiency decreased the population of the pathogenic CD4 T_EM_ cells and restored the expression of 15-PGDH in colon epithelial cells, both events that are regulated by TGF-β. These results are consistent with previous reports that TGF-β signaling *via* SMAD3 inhibits IFN-γ production (51) and IFN-γ/STAT pathway inhibits TGF-β/SMAD pathway (52).

To our knowledge, this is the first report describing dual, tandem mechanisms for tumor promotion by IFN-γ, that include the expansion of pathogenic effector CD4^+^ T cells within the tumor microenvironment (TME) and concomitant suppression of 15-PGDH expression in epithelial cells. Importantly, strategies designed to modulate the expression and activity of soluble factors in both the epithelial and stromal compartment in the TME may serve to concomitantly support the maintenance of mucosal epithelial homeostasis and suppress the expansion of pathogenic, tissue-resident effector T cells producing inflammatory cytokines, thereby forming a unique and effective approach to cancer immunotherapy.

## Data availability statement

The original contributions presented in the study are included in the article/**Supplementary Material**, further inquiries can be directed to the corresponding author.

## Ethics statement

The animal study was reviewed and approved by The Institutional Animal Care and Use Committee at Case Western Reserve University.

## Author contributions

SC, JL, and B-GK designed the studies and developed the methodology; SC and B-GK performed experiments; SC, AH, JL, and B-GK interpreted the data; SC and B-GK wrote the initial draft of the manuscript; SC, AH, JL, and B-GK edited the manuscript and approved the final version of the manuscript. All authors contributed to the article and approved the submitted version.

## Funding

This work was supported by the National Institutes of Health grants (R01CA168586 and 1R03CA259901-01A1).

## Conflict of interest

The authors declare that the research was conducted in the absence of any commercial or financial relationships that could be construed as a potential conflict of interest.

## Publisher’s note

All claims expressed in this article are solely SFthose of the authors and do not necessarily represent those of their affiliated organizations, or those of the publisher, the editors and the reviewers. Any product that may be evaluated in this article, or claim that may be made by its manufacturer, is not guaranteed or endorsed by the publisher.
