# Supplementary material for: Smad4-deficient T cells promote colitis-associated colon cancer via an IFN-γ-dependent suppression of 15-hydroxyprostaglandin dehydrogenase
Source: Front Immunol. 2022 Aug 15;13:932412. doi: 10.3389/fimmu.2022.932412 (PMC9420841; doi:10.3389/fimmu.2022.932412)
Supplement: Supplementary file 1 [file Presentation_1.pptx]

## Slide 1
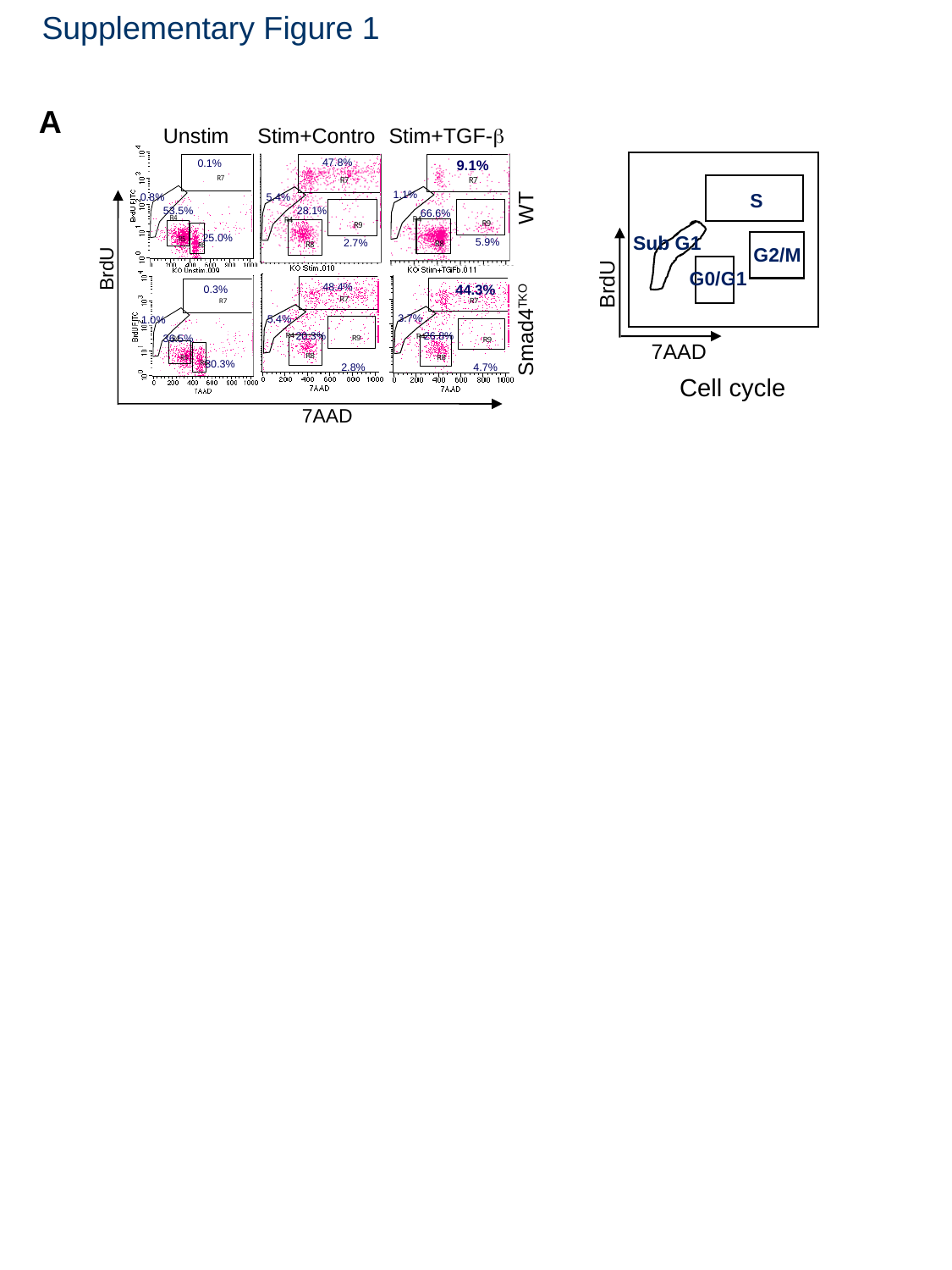

Supplementary Figure 1
A
Stim+TGF-b
Unstim
Stim+Control
47.8%
0.1%
9.1%
1.1%
0.8%
5.4%
53.5%
28.1%
66.6%
25.0%
5.9%
2.7%
BrdU
48.4%
44.3%
0.3%
3.7%
5.4%
1.0%
20.3%
26.8%
36.5%
30.3%
2.8%
4.7%
7AAD
WT
Smad4TKO
S
Sub G1
G2/M
G0/G1
BrdU
7AAD
Cell cycle

## Slide 2
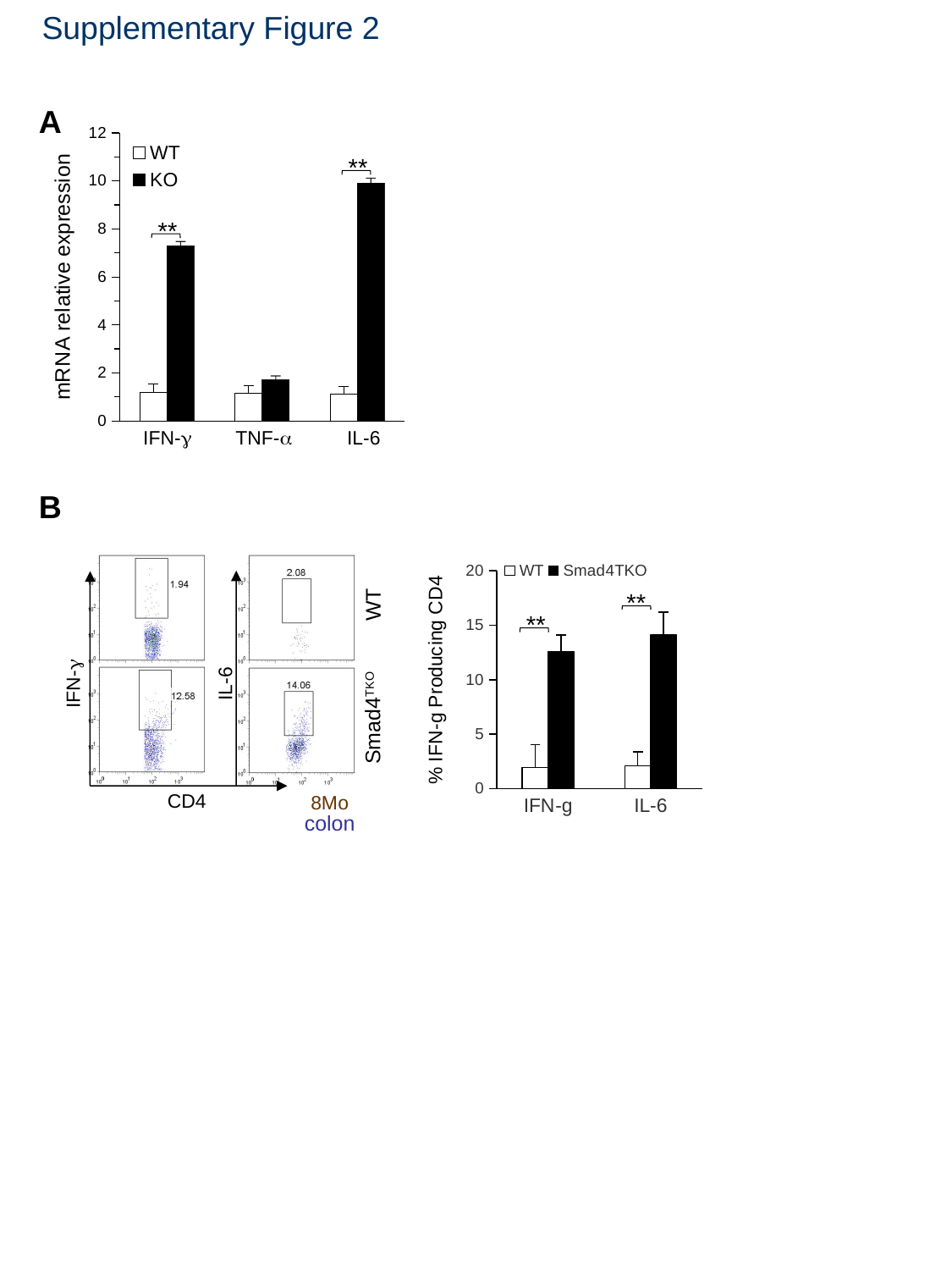

Supplementary Figure 2
A
### Chart
| Category | WT | KO |
|---|---|---|IFN-g TNF-a IL-6
**
**
B
### Chart
| Category | WT | Smad4TKO |
|---|---|---|
| IFN-g | 1.94 | 12.6 |
| IL-6 | 2.1 | 14.1 |**
**
WT
IL-6
IFN-g
Smad4TKO
CD4
8Mo
colon

## Slide 3
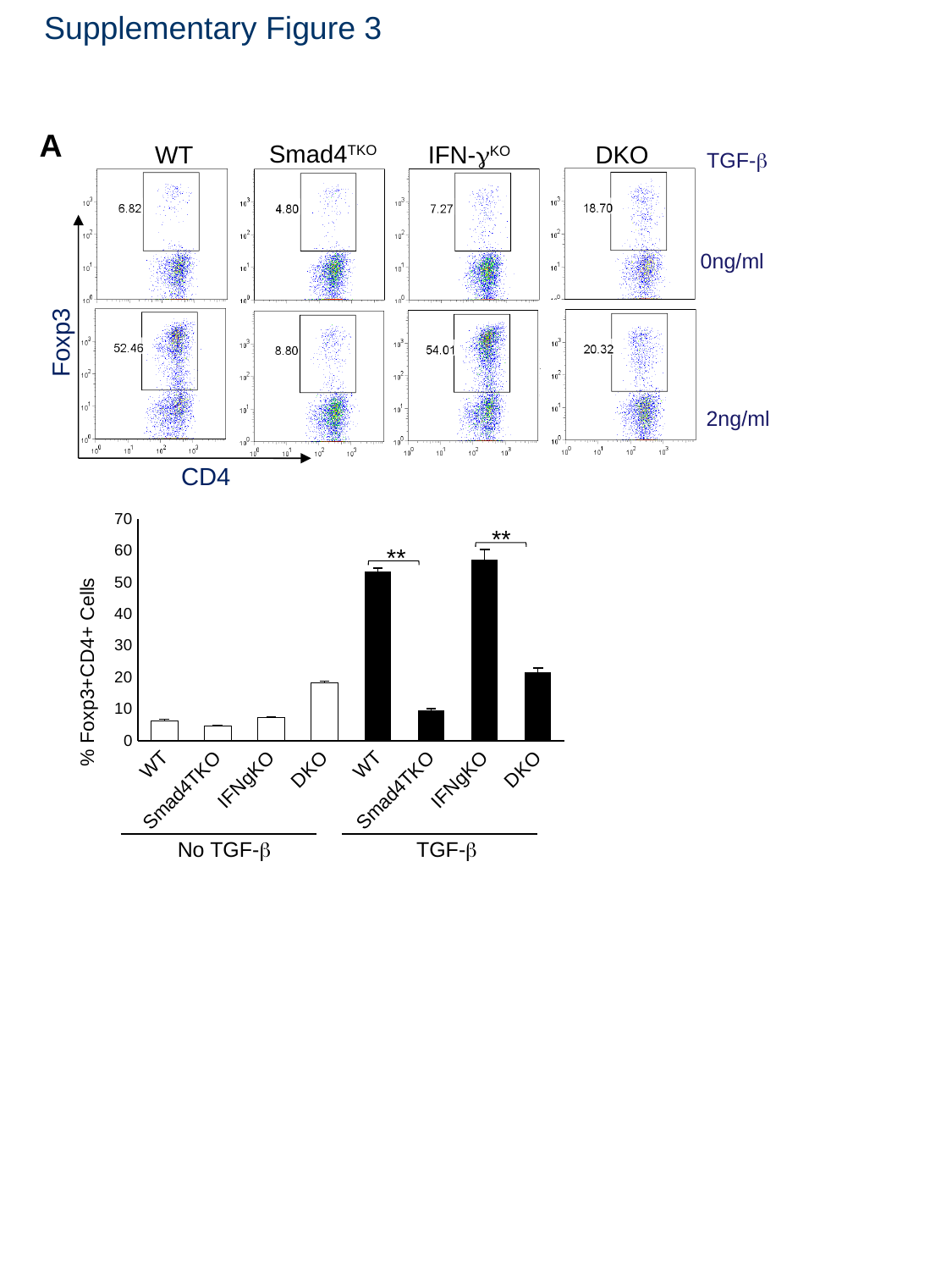

Supplementary Figure 3
A
Smad4TKO
IFN-gKO
DKO
WT
TGF-b
0ng/ml
Foxp3
2ng/ml
CD4
### Chart
| Category | |
|---|---|
| WT | 6.386666666666668 |
| Smad4TKO | 4.703333333333333 |
| IFNgKO | 7.383333333333333 |
| DKO | 18.253333333333334 |
| WT | 53.47666666666667 |
| Smad4TKO | 9.493333333333334 |
| IFNgKO | 57.196666666666665 |
| DKO | 21.620000000000005 |**
**
No TGF-b
TGF-b

## Slide 4
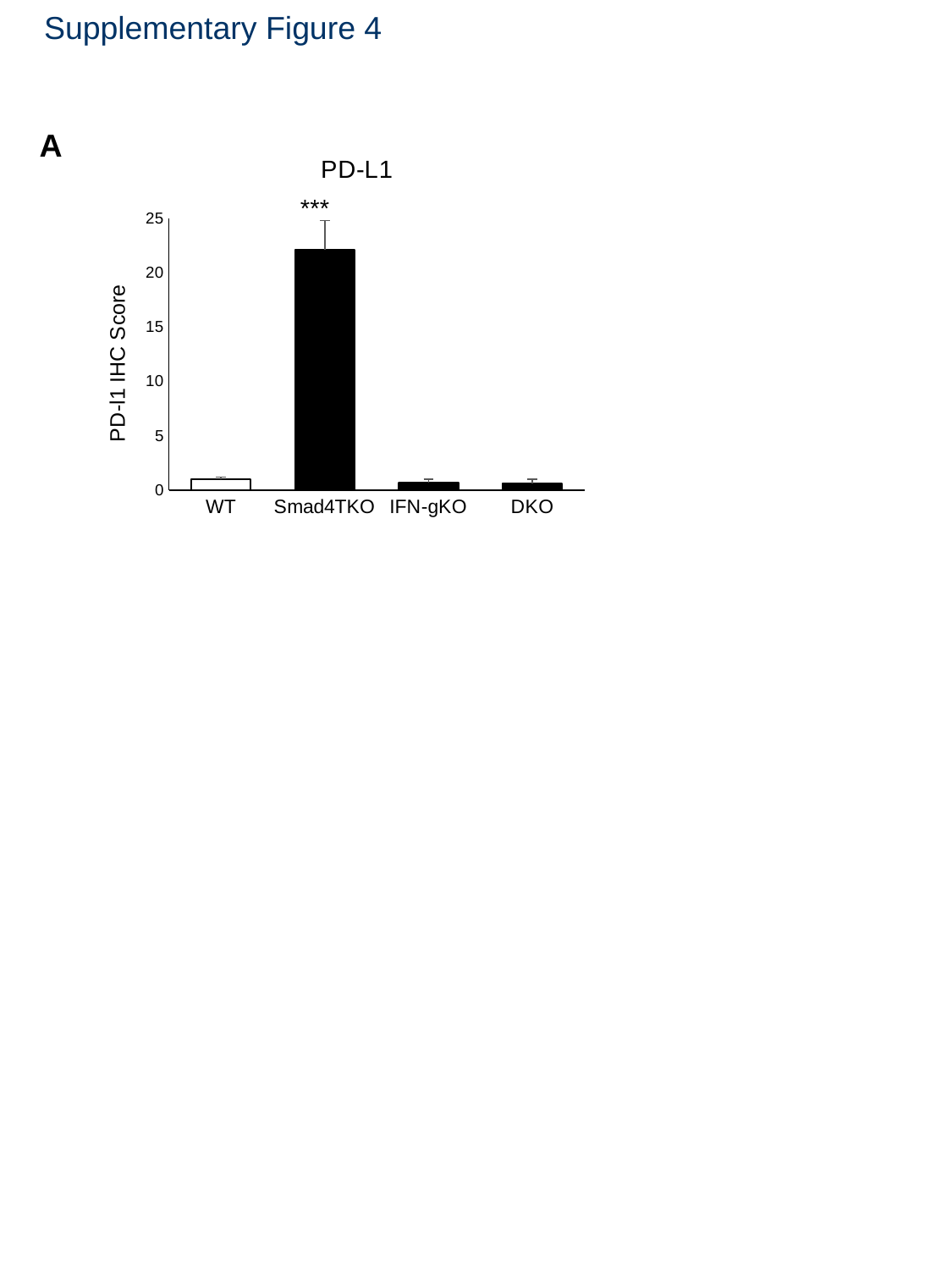

Supplementary Figure 4
A
### Chart: PD-L1
| Category | |
|---|---|
| WT | 1.0 |
| Smad4TKO | 22.1 |
| IFN-gKO | 0.7 |
| DKO | 0.6 |***
